# Minimally Invasive Standalone Left Atrial Appendage Occlusion for Atrial Fibrillation: Procedural Approaches and Complications

**DOI:** 10.3390/jcm15145587

**Published:** 2026-07-16

**Authors:** Sandra Jaksic Jurinjak, Vlatka Reskovic Luksic, Tomislav Kopjar, Vedran Velagic

**Affiliations:** 1 Department of Cardiovascular Diseases, University Hospital Centre Zagreb, Kispaticeva 12, 10000 Zagreb, Croatia; 2 School of Medicine, University of Zagreb, Salata 3, 10000 Zagreb, Croatia; 3 Algebra Bernays University, Gradiscanska 24, 10000 Zagreb, Croatia; 4 Department of Cardiac Surgery, University Hospital Centre Zagreb, Kispaticeva 12, 10000 Zagreb, Croatia

**Keywords:** left atrial appendage, occlusion, minimally invasive procedure

## Abstract

The left atrial appendage is well recognized as the site of thrombus formation in patients with atrial fibrillation. However, in patients who are either unsuitable for long-term oral anticoagulation or in whom this therapy is inefficient, left atrial occlusion has emerged as a mechanical strategy option to diminish stroke risk. Minimally invasive percutaneous and standalone surgical thoracoscopic techniques are appearing as viable options for left atrial appendage exclusion, each with distinct procedural risk profiles and characteristics, as well as evidence from trials or registers. We suggest that the choice between percutaneous and thoracoscopic left atrial appendage occlusion should be individualized, ideally within the multidisciplinary heart team, considering left atrial appendage anatomy, patient bleeding and thromboembolic risk profile, comorbidities, prior cardiac interventions, and institutional expertise and resources. We aim to present in this review the value of multimodality imaging in patient selection for minimally invasive left atrial appendage occlusion to minimize the possibility of complications, and to compare technical advancements and indications for percutaneous and standalone thoracoscopic left atrial appendage occlusion.

## 1. Introduction

Atrial fibrillation (AF) is the most frequently encountered arrhythmia, carrying with it a substantially increased risk of stroke, up to five-fold, which significantly impacts morbidity and mortality [1,2,3,4]. The left atrial appendage (LAA) is frequently identified as the site of thrombus formation in nonvalvular atrial fibrillation patients [1]. Therefore, occluding the left atrial appendage with the device has recently emerged as a strategy in stroke prevention for individuals with atrial fibrillation, particularly those for whom long-term anticoagulation is not a viable option [2,3]. Long-term anticoagulation therapy, while effective, carries a significant risk, with bleeding as the most concerning complication [2,3]. Anticoagulant treatment for atrial fibrillation, and especially the introduction of new dual anticoagulant drugs as a therapeutic option (DOACs), led to a new era of prevention of stroke in AF patients [5]. However, DOAC therapy is also not free of complications; therefore, there remains a subgroup of patients who have intolerance to or have experienced ineffectiveness of anticoagulant therapy, which is an on-going challenge in managing AF patients [5,6]. The pursuit of effective left atrial appendage occlusion has led to the development of both percutaneous and surgical thoracoscopic techniques or surgical techniques in combination with other indications for cardiac surgery, each with its unique set of advantages and disadvantages [3,4]. Percutaneous left atrial appendage occlusion (pLAAO) involves the deployment of a device within the LAA via catheter, typically introduced through the femoral vein. This minimally invasive approach offers the benefit of avoiding major surgery, potentially leading to shorter recovery times and reduced hospital stays. Percutaneous or minimally invasive standalone thoracoscopic method LAA occlusion (tLAAO) can also be an alternative to anticoagulation in AF patients. Traditionally, surgical LAAO is mostly additional procedure to other open heart surgery procedures or as an addition to Cox Maze procedures for AF, and also serves for patient risk reduction in ischemic stroke [7,8,9,10,11,12]. In that case of combined surgery, ACC/AHA/ACCP/HRS (American College of Cardiology, American Heart Association, Heart Rhythm Society) guidelines recommend percutaneous LAAO with the I class of recommendations [12]. On the other hand, percutaneous and standalone thoracoscopic LAAO is recommended with the IIb class of recommendations for patients with AF and contraindication to long-term anticoagulation in the current ESC (European Society of Cardiology) guidelines developed in collaboration with the European Association for Cardio-Thoracic Surgery (EACTS) [7,8,13]. Additionally, ACC/AHA/ACCP/HRS (American College of Cardiology, American Heart Association, Heart Rhythm Society) guidelines recommend percutaneous LAAO with the IIa class of recommendations [12]. It should be noted, AF accounts for up to 25% of ischemic strokes; therefore, other factors, such as atherosclerosis, arterial hypertension, and dyslipidemia, should always be considered and treated according to the best of knowledge and current optimal medical therapy [8,13]. There is increasing evidence of better prognosis after LAAO; however, potential downsides, such as incomplete sealing of the LAA or device-related thrombus, remain a concern [8,13]. LAAO patient selection needs to be guided by the best and most safe outcome for selected patients; therefore, in this setting and this patient subgroup, multimodality imaging is paramount.

In presenting illustrative clinical cases, we aim to demonstrate the imaging-based heart team patient selection for minimally invasive LAAO, and to compare technical advancements and indications, as well as possible complications of percutaneous and standalone thoracoscopic left atrial appendage occlusion.

### 1.1. Percutaneous LAAO

**Figure 1:**
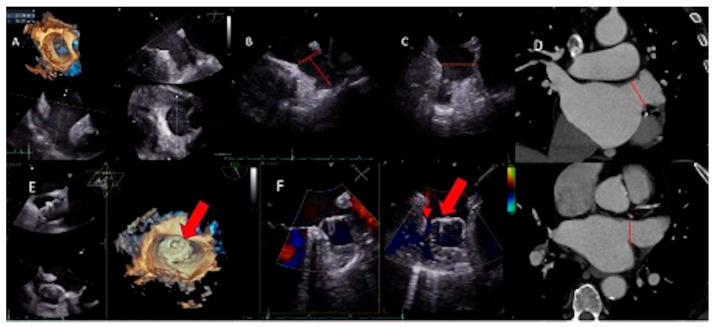
(**A**) 3D TEE reconstruction of LAA, (**B**) 2D TEE measurements of LAA orifice mid esophageal position 90° (orifice marked with a thin red line), (**C**) 2D TEE measurements of LAA orifice mid esophageal position 135° (orifice marked with a thin red line), (**D**) CCT measurements of LAA orifice (marked with a thin red line), (**E**) 3D TEE of device position in LAA (red arrow showing the device), (**F**) small PDL before repositioning of the device (red arrow showing the device placed in the LAA and to left PDL of 3 mm- marked with a thin red arrow).

Left atrial appendage (LAA) occlusion is indicated for several patient populations, particularly those with atrial fibrillation (AF). Key indications for percutaneous LAAO based on the current ESC guidelines are indicated for patients with AF and contraindications for long-term anticoagulant treatment to prevent ischemic stroke and thromboembolism, or may be considered for stroke prevention in patients with AF who have contraindications for long-term anticoagulation, particularly in those with a life expectancy greater than 12 months [7,8,9]. First devices for percutaneous LAAO were introduced in the 2000s, and since then, studies as well as registries have shown the procedure to be successful and safe even in low volume centers, leading to the conclusion that it could be the method of treatment in selected patients [10,11,12,14]. Systems most used in Europe, as well as in our institution, are Watchman FLX (Boston Scientific, Marlborough, MA, USA) and Amplatzer Amulet (Abbott, Green Oaks, IL, USA). The Watchman device is shaped as a parachute with 12 fixation anchors to secure the stability of the device [10]. The selected size should be from 10 to 30% larger than the measured dimension of LAA. The Watchman FLX and FLX Pro devices are available in sizes from 20 to 40 mm, respectively [10]. The Amplatzer Amulet is a dual seal device where the disk is connected to the lobe, made of nitinol mesh, and comes in sizes from 16 to 34 mm [14,15]. Because the morphology or anatomy of the LAA is widely diversiform and variable, attempts were made to classify the morphology in different imaging modalities. The LAA has been classified by multimodality imaging into four types based on its anatomy: cactus, cauliflower, windsock, and chicken wing [16]. Pre-interventional multimodality imaging is therefore crucial in deciding the interventional plan [16]. Proper device-choosing and device-sizing, as well as excluding contraindications for implantation, are based on transesophageal (TEE) echocardiography, as the standard method in assessing the patients for LAAO candidacy; however, morphology assessment can prove to be challenging with TEE. Further expanding imaging by adding the cardiac computed tomography (CCT) is allowing more accurate differentiation of LAA morphological types as presented in illustrative case in Figure 1 [17].

For illustration of the screening process and LAAO strategy selection, we present the case of a male patient with atrial fibrillation and a contraindication to long-term anticoagulation because of recurrent severe urinary tract hemorrhages. Preprocedural evaluation was performed using both transesophageal echocardiography (TEE) and CCT, enabling comprehensive characterization of the LAA anatomy. In presented case, the LAA exhibited a cauliflower morphology with multiple secondary lobes, visualized on three-dimensional reconstruction (Figure 1A) and corresponding 2D TEE views (Figure 1B,C). Appropriate device selection and procedural planning depends on the specific LAA morphology and dimensions, therefore detailed anatomical assessment is essential. Measuring the LAA orifice diameter and depth is crucial in the process of device selection. Sizing is done in 2D TEE in mid esophageal views at 0°, 45°, 90°, and 135° (Figure 1B,C). If the quality of images is sufficient, the quicker and easier method is the 3D reconstruction of the LAA orifice and the landing zone for the device (Figure 1A). However, TEE has limited accuracy in the visualization of the entire LAA anatomy and side lobes. Therefore, in planning the procedure, CCT is of great value, particularly in assessing the anatomy of the LAA and the sizing of the device (Figure 1D) [17]. The circumflex artery and the left superior pulmonary vein are main landmarks necessary for proper orientation of the device in relation to the surrounding structures. Although TEE is excellent imaging modality for the detection of thrombus in LAA, possible false positive thrombus detections have been reported. Therefore, CCT imaging provides crucial information, with high sensitivity and negative predictive value [17,18]. Specificity of CCT can be further improved by completing the diagnostic search adding delayed acquisition at a 6 min delayed phase to the angiographic phase [19]. CCT can be an alternative in patients with relative contraindication for TEE, although it cannot guide the procedure. In this case, ICE (intracardiac echo) can be used as an additional method of imaging [17,19,20]. During the procedure, TEE is also used to guide septal puncture and guide the system towards LAA, as well as detecting possible thrombus in real time [17,21]. In the case we present, based on the anatomy and sizing (23.8 mm × 30.3 mm) of the LAA we finally decided to implant the Watchman device size 34 mm (Figure 1E), at the time of the intervention largest size of the device, with concerns regarding the landing depth. After repositioning because of the small 3 mm PDL (peri-device leak), the position was accepted (Figure 1F—red arrow showing the device placed in the LAA and to left PDL of 3 mm), and in follow-up, there is no DRT (device-related thrombus), which is known to be found if LAA has not been fully occluded [22]. Patient is on reduced anticoagulation dose (apixaban 2.5 mg twice a day). Newly introduced large devices (40 mm) can be an option for percutaneous LAAO; however, the concern of acceptable depth for large device accommodation and unfavorable anatomy remains.

### 1.2. Standalone Thoracoscopic LAAO

The surgical epicardial approach of LAAO can be achieved by excision, exclusion by suture, or exclusion with a device. The family of AtriClip devices (AtriCure Inc., Mason, OH, USA) is the most widely used LAAO device for surgical occlusion of the LAA [23]. In the recent guidelines, standalone thoracoscopic LAAO (tLAAO) is recommended with the IIb class of recommendations for patients with AF and contraindication to long-term anticoagulation [8,12]. Percutaneous LAAO holds an equal class of recommendation for the same indication in current ESC (European Society of Cardiology) guidelines developed in collaboration with the European Association for Cardio-Thoracic Surgery (EACTS) [8]. Each of these approaches have their advantages and disadvantages.

Left atrial appendage with complex anatomy, large LAA with multiple lobes and complex take-offs or proximal lobes, are not suitable for percutaneous LAAO [24,25,26,27]. These anatomy variations can be associated with suboptimal positioning of the device, possibly leading to DRT and PDL, as well as device embolization. In addition to the standard patients with OAC contraindication, these patients unsuitable for percutaneous LAAO can be candidates for minimally invasive (standalone) thoracoscopic LAAO. Anatomical variations in the LAA are not a matter of concern for epicardial LAAO.

One of the main advantages of thoracoscopic over transcatheter LAAO is the epicardial nature of the approach. Since the device is not placed in the bloodstream, it does not require any antithrombotic prophylaxis. Due to its epicardial positioning, DRT is not a matter of concern. Thoracoscopic standalone epicardial LAA closure in patients with a contraindication for anticoagulation therapy may take precedence over the percutaneous approach because there is no need for post-procedure anticoagulation after epicardial closure due to almost full exclusion of the LAA and thus lesser thromboembolic events [23,26,27,28]. Compared with conventional median sternotomy, the thoracoscopic approach offers the advantages of reduced surgical trauma, faster recovery, and improved cosmetic outcomes. However, thoracoscopic procedures are technically more demanding, require specialized equipment and expertise, and may be associated with steeper learning curve, particularly during the initial adoption phase.

Interestingly, placement of the AtriClip device also causes electrical disactivating of the LAA in AF patients [23]. Data show that standalone LAA closing with an epicardial clip is safe [27,29,30,31]. Safety data and experience in epicardial LAA closure originate mostly from a single clipping device (AtriClip) [27]. There are no large RCTs directly comparing percutaneous vs. thoracoscopic LAA occlusion; nevertheless, available observational data suggest that thoracoscopic AtriClip may achieve more complete exclusion with fewer leaks; whereas, percutaneous devices have easier recovery but require at least short-term anticoagulation [23,27,32,33,34,35,36].

We suggest that the heart team, consisting of an interventional or EP cardiologist, clinical/imaging cardiologist, and cardiothoracic surgeon, should, therefore, choose between percutaneous or epicardial thoracoscopic exclusion by device. The decision should be based on clinical features of the patient as well as the anatomy of LAA assessed by multimodality imaging. The choice between percutaneous and thoracoscopic LAAO should be individualized based on patient characteristics, including left atrial appendage anatomy, comorbidities, bleeding risk, surgical risk, and the presence of concomitant cardiac conditions requiring surgery (Table 1).

**Figure 2:**
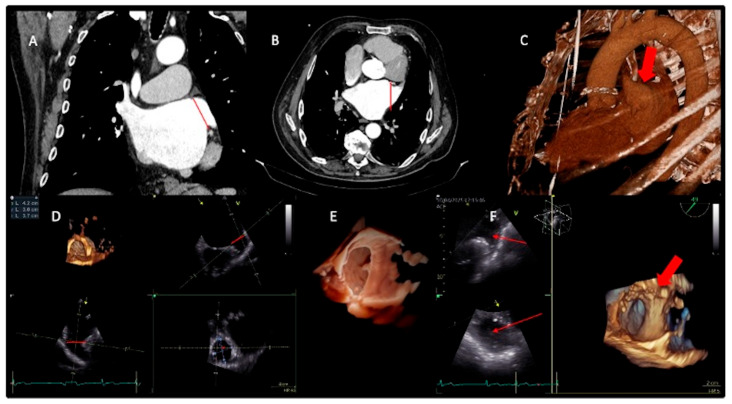
(**A**,**B**) CCT showing wide and shallow LAA (LAA orifice marked with a thin red line). (**C**) 3D CT reconstruction of LAA (red arrow pointing to the LAA). (**D**–**F**) 3D TEE reconstruction of large and shallow LAA (dimension of orifice 42 mm × 37 mm) (LAA orifice marked with a thin red line, red arrow marking the LAA).

Proper device sizing and excluding contraindications for implantation based on transesophageal (TEE) and multimodality imaging, mainly cardiac computed tomography (CCT), is crucial in procedure planning for percutaneous LAAO device placement. Herein, we present illustrative case Figure 2. CCT (Figure 2A–C) and TEE (Figure 2D–F) 2D and 3D imaging for percutaneous LAAO procedure planning. The patient had a history of cerebral hemorrhage, persistent AF, thus contraindication for oral anticoagulation. Both imaging techniques have shown wide and shallow (42 mm × 37 mm) cauliflower-shaped LAA. The heart team decided that the optimal and safest approach in this patient would be thoracoscopic LAAO with the AtriClip device. Furthermore, after implantation, all anticoagulation was stopped, and the patient is well and in follow-up.

### 1.3. LAAO Complication Management

**Figure 3:**
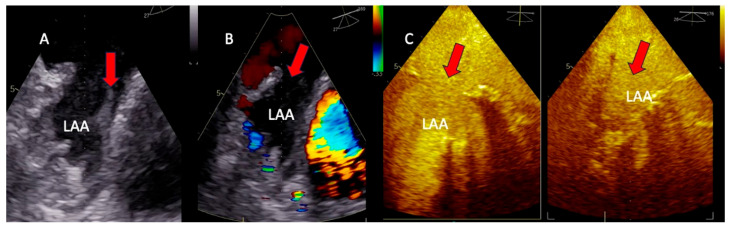
(**A**) TEE suspected thrombus in LAA (red arrow marking the thrombus), (**B**) TEE with Color Doppler showing poor function of LAA (red arrow), (**C**) contrast opacification of LAA for thrombus exclusion (red arrow pointing to LAA).

While there are clear benefits of LAAO, there are still challenges with occlusion of the LAA as a periprocedural complication, peri-device leak (PDL), device embolization, and device-related thrombosis (DRT) [18,37]. The most common periprocedural complication is pericardial effusion, but air embolism and periprocedural stroke can also occur. Device-related thrombosis (DRT) may occur due to device or procedure-related factors or postprocedural antithrombotic [18,37]. Exclusion of the presence of thrombus is paramount before implanting the device and is enabled by TEE in planning the procedure and during the procedure. TEE is of great importance in decision-making in real time by imaging in 2D, 3D, as well as assessing the LAA with the Color Doppler in multiplanes. In case of inconclusive findings, the contrast opacification (Figure 3. Optison- Microspheres Injectable Suspension, GE, USA) of LAA can exclude the presence of thrombus by opacification the left atrial appendage as shown in Figure 3. Musculi pectinate, as varieties of anatomical forms that can be found in the LAA, can sometimes differentially appear as thrombi [21,38,39,40].

**Figure 4:**
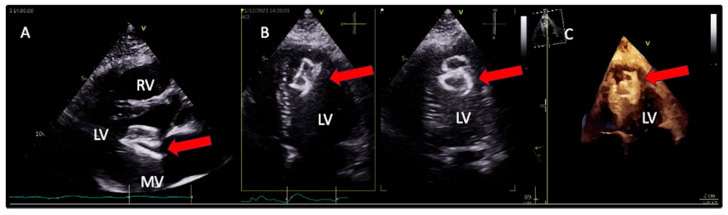
(**A**) TTE (PLAX view) showing embolization of the device (red arrow) at the level of the mitral valve, (**B**) embolization of the device in the apex of the left ventricle (red arrow marking the device), (**C**) embolization of the device in the apex of the left ventricle in 3D TTE (focus 4CH view) (red arrow).

A peri-device leak (PDL) is described in epi and endocardial LAAO, therefore, it is paramount to address modifiable risk factors, as device-sizing and compression, as well as device orientation in the LAA. One of the most serious complications is device embolization (Figure 4). Data from the registries report the incidence from 0.07 to 0.7% [37,41,42,43].

Here, we present a case of device embolization in a female patient with paroxysmal AF and a contraindication to oral anticoagulation due to a previous cerebral hemorrhage, who underwent pLAAO (device type Amplatzer Amulet LAAO, Abbott Medical Inc., North Chicago, IL, USA). Perioperatively, at the last assessment of the completed procedure, the tug-test for anchoring on TEE, as well as DSA (digital substraction angiography), confirmed adequate device positioning and compression. However, approximately 10 h after the procedure, significant ventricular extrasystole had occurred. The patient was asymptomatic. Transthoracic echocardiography (TTE) showed the device in the position of the mitral valve traveling to the apex of the left ventricle (Figure 4A–C, Videos S1 and S2). The patient was referred to surgery for device extraction and endocardial LAAO and is doing fine in follow-up.

**Table 1:** Main characteristics of percutaneous and standalone thoracoscopic LAAO approaches (pLAAO—percutaneous; tLAAO—standalone thoracoscopic LAAO; LAAO—left atrial appendage occlusion; TEE—transesophageal echocardiography; RTC—randomized controlled trial) [7,8,9,10,11,12,13,14,19,23,26,27,31,32,33,34,35,36,37,41,44].

Parameters	pLAAO	tLAAO
**Approach**	Transseptal catheter via femoral vein	Video-assisted thoracoscopic surgery
**Typical Candidates**	Unable to tolerate long-term anticoagulation	Unable to tolerate long-term anticoagulation
**Preferred When**	Minimally invasiveness and rapid recovery are priorities	Anatomy unsuitable for percutaneous approach
**Procedural success**	95–98% (experienced operators)	>95% with proper technique
**Stroke Prevention**	Non-inferior to warfarin (PROTECT AF, PREVAIL)	Effective; supported mainly by observational studies
**Residual Leak**	Small leaks possible; (>5mm leak <1%	Rare; usually complete exclusion
**Periprocedural Stroke**	Very low	Very low
**Pericardial** **Effusion/Tamponade**	1–2%	<1–2%
**Device Embolization**	Rare (<1%)	Not applicable
**Bleeding**	Lower overall risk	Higher immediate surgical risk
**Infection**	Very low	Possible surgical-site infection
**Post-Procedure** **Antithrombotic Therapy**	Short-term anticoagulation or antiplatelet therapy usually	Often unnecessary if complete exclusion achieved
**Long-Term** **Anticoagulation**	Mostly not required after device endothelization	Not required if exclusion remains complete
**Advantages**	Minimally invasive, short recovery	No need for anticoagulation; complete appendage exclusion
**Limitations**	Not suitable for all anatomies; may require temporary anticoagulation	More invasive, higher cost, limited expertise availability

## 2. Discussion

LAA is well recognized as the site of thrombus formation in patients with non-valvular atrial fibrillation (AF), accounting for over 90% of cardiac emboli in this population [1,2,3,4,31,32]. LAA occlusion, as a mechanical strategy to diminish stroke risk, has emerged as a therapeutic option in patients who are either unsuitable for or intolerant to long-term oral anticoagulation. Both percutaneous and surgical thoracoscopic techniques have emerged as viable options for LAA exclusion, each with distinct procedural risk profiles and characteristics, as well as evidence from trials or registers [8,9,10,11,12,13,14,23,27,32,33,34,35,36]. The percutaneous approach is now a well-established method, following multiple randomized controlled trials. The PROTECT AF trial, conducted between 2005 and 2008, was the first pivotal randomized study to establish percutaneous LAAO as a viable alternative to long-term warfarin therapy, leading towards broader clinical adoption of the technique. The Watchman device in the PROTECT AF trial was shown to be non-inferior to warfarin for the composite endpoint of stroke, systemic embolism, and cardiovascular death, but with a higher rate of periprocedural complications, notably pericardial effusion, and device embolization [31]. Subsequent studies, including PREVAIL (2010–2012), ASAP (2009–2011), and Amulet IDE (2016–2020), further refined patient selection, procedural safety, and post-procedural antithrombotic management, expanding role of percutaneous LAAO in contemporary stroke prevention strategies [32,33,34]. The PREVAIL trial demonstrated improved procedural safety and confirmed non-inferiority with updated implantation techniques [32]. Additionally, the data provided in ASAP registry evidence supported off-label use of the Watchman device in patients with contraindications to oral anticoagulation, using dual antiplatelet therapy post-procedure in stroke risk reduction [33]. Direct comparison between the two most used devices (Amulet IDE trial) demonstrated comparable efficacy in stroke prevention, with the Amulet device showing fewer peri-device leaks but a higher rate of procedure-related complications [34]. Advanced techniques are also emerging, such as the introduction of a steerable delivery sheath, resulting in a lower incidence of residual patent LAA, off-axis device position, and PDL due to better LAA sealing [35,36]. Furthermore, new emerging evidence are showing (OPTION trial) that, among patients who underwent catheter-based atrial fibrillation ablation, LAAO can lower risk of non-procedure related major or clinically relevant nonmajor bleeding compared to oral anticoagulation and showing noninferiority to oral anticoagulation regarding a composite of death from any cause, stroke, or systemic embolism; therefore, this will probably lead to more trials and data in the near future [44,45,46,47]. Recent evidence from the OPTION Trial has renewed interest in the broader role of left atrial appendage occlusion (LAAO) as an alternative to long-term oral anticoagulation in selected patients with atrial fibrillation [44]. By demonstrating that LAAO performed in conjunction with catheter ablation can provide comparable protection against thromboembolic events while reducing bleeding risk, the trial has strengthened the rationale for integrating stroke prevention strategies directly into rhythm control interventions. This concept may be particularly relevant in patients in whom lifelong anticoagulation is associated with substantial risk, such as frail elderly patients. Although frailty is not currently incorporated into conventional stroke-risk scores, it can frequently influence clinical decision making. Frail individuals often have a high thromboembolic risk and are exposed to increased risks of falls, bleeding, polypharmacy, and treatment nonadherence. Future studies should investigate whether an imaging-guided intervention combined with risk stratification approach can identify frail patients who may benefit more from LAAO than from continued anticoagulation, especially considering quality of life and functional independence. Patients with a history of intracranial hemorrhage, as in our case, constitute another subpopulation of frail patients. Robust randomized data in this subpopulation remain limited; however, LAAO offers an attractive non-pharmacological strategy for stroke prevention. This strategy may reduce cumulative procedural burden, and potentially minimize long term exposure to anticoagulation. Therefore, advances in preprocedural imaging, three-dimensional imaging reconstruction and patient specific anatomical and clinical assessment may facilitate more individualized selection of patients suitable for concomitant intervention [44,45,46,47,48].

Thoracoscopic access LAA exclusion (mainly AtriClip device) provides an alternative pathway with certain anatomical and clinical advantages. The procedure allows direct visualization and mechanical closure of the LAA at its base, which has been shown to result in durable and complete exclusion with minimal risk of residual flow [29,30]. Thoracoscopic LAA exclusion has been increasingly adopted as a standalone procedure in patients with contraindications to anticoagulation or previous failed percutaneous closure. In addition, surgical left atrial appendage occlusion is performed concomitantly with cardiac surgery for other indications, such as valve or coronary artery procedures, in the majority of patients. However, standalone thoracoscopic intervention in selected patients may be limited by the availability of specialized equipment and expertise, as well as operating room availability. Observational studies and multicenter registries have shown high procedural success rates, often exceeding 95%, with low complication rates and residual leaks (with AtriClip typically complete exclusion) when performed in experienced centers [30,31,37]. Unlike percutaneous devices, the AtriClip does not reside within the cardiac chamber, eliminating the risk of device-related thrombosis and avoiding the need for post-procedure anticoagulation in many cases [27]. The data support the stroke reduction benefit of surgical LAA closure as a class I recommendation, at the time of cardiac surgery, significantly reducing the risk of stroke or systemic embolism compared to no occlusion, without increasing perioperative risk (LAAOS III trial) [12,29,43]. However, regardless of conventional cardiac surgery, stand-alone thoracoscopic LAAO is recommended with the IIb class of recommendations for patients with AF and contraindication to long-term anticoagulation [8,12,43]. Nevertheless, thoracoscopic stand-alone LAA occlusion is more invasive compared to percutaneous, typically requiring general anesthesia and thoracic access, which may be contraindicated in patients with severe pulmonary disease, prior thoracic surgery, or significant pleural adhesions [12,20,43]. The requirement for surgical expertise and dedicated resources may also limit its availability in some centers. Percutaneous techniques offer the advantage of being minimally invasive, with most procedures performed under conscious sedation. Recovery is generally swift, making the procedure suitable for elderly or frail patients. However, successful device deployment is highly dependent on LAA morphology, residual peri-device leaks are not uncommon, and most protocols necessitate short-term antithrombotic therapy, which may be challenging in patients with absolute contraindications to any form of anticoagulation or antiplatelet therapy [37,47,49].

We propose that the choice between percutaneous and thoracoscopic standalone LAAO should be individualized, ideally within the multidisciplinary heart team, considering LAA anatomy, patient bleeding and thromboembolic risk profile, comorbidities, prior cardiac interventions, and institutional expertise and resources (Table 1). Percutaneous LAAO may be preferred in patients at high surgical risk or with favorable appendage anatomy; whereas, thoracoscopic LAAO may be considered in patients unsuitable for transcatheter closure, those seeking complete epicardial exclusion, or when concomitant surgical treatment is planned. Future research should also evaluate whether integrated ablation and LAAO strategies improve long-term outcomes in high-risk populations, particularly those with elevated bleeding risk, frailty, or prior intracranial hemorrhage. Ultimately, the future of LAAO is likely to move beyond a simple alternative for patients with contraindications to anticoagulation toward a personalized, imaging guided therapeutic strategy, incorporating risk profiling, for more precise identification of patients who will derive the benefit from either percutaneous or thoracoscopic LAAO.

## 3. Conclusions

Percutaneous and thoracoscopic standalone LAA occlusion techniques are both viable; however, there is a lack of direct comparative studies between the two and direct comparative data are limited. Percutaneous devices are supported by multiple randomized controlled trials and have become the standard of care for most patients with AF who are poor candidates for anticoagulation. On the other hand, thoracoscopic LAA occlusion offers superior anatomical exclusion and likely no need for post-procedure antithrombotic or anticoagulation therapy, although it involves a more complex process. It is important to note that the best approach for individual patients in clinical contexts, as is yet to be defined in randomized comparison, for now should be made within the multidisciplinary heart team in order to achieve maximum safety and reduce complications in these subgroups of patients.

## Data Availability

The original contributions presented in this study are included in the article/Supplementary Materials. Further inquiries can be directed to the corresponding author.

## Supplementary Materials

The following supporting information can be downloaded at: https://www.mdpi.com/article/10.3390/jcm15145587/s1. Video S1. TTE PLAX (parasternal long axis) view of device embolization at mitral position (supplement). Video S2. TTE 4CH view of device embolization at apex of the left ventricle (supplement).
